# *Chrysanthemum lavandulifolium* Essential Oil Attenuates Periodontitis via Antibacterial and Anti-Inflammatory Effects

**DOI:** 10.3390/ijms27135966

**Published:** 2026-07-02

**Authors:** Juan Ma, Likuan Liu, Yi Ren, Mingjin Wang, Xing Li, Jinping Li

**Affiliations:** 1 Qinghai Provincial Key Laboratory of Medicinal Plant and Animal Resources of Qinghai–Tibet Plateau, School of Life Sciences, Qinghai Normal University, Xining 810008, China; juan_ma0522@163.com (J.M.); 2025105@qhnu.edu.cn (L.L.); 15029332699@163.com (Y.R.); wmj8598@163.com (M.W.); lx20242024@126.com (X.L.); 2 Academy of Plateau Science and Sustainability, Qinghai Normal University, Xining 810008, China

**Keywords:** *Chrysanthemum lavandulifolium* essential oil (CLEO), periodontitis, *Porphyromonas gingivalis*, molecular docking, network pharmacology, inflammatory factors

## Abstract

Periodontitis, driven by *Porphyromonas gingivalis* (*P. gingivalis*) biofilms, is a global health burden with limited treatment options due to antibiotic resistance. *Chrysanthemum lavandulifolium* is traditionally used in China for clearing heat and reducing swelling, yet its anti-periodontitis potential remains uncharacterized. This study evaluated the antibacterial and therapeutic effects of its essential oil (CLEO) against periodontitis. The minimum inhibitory concentration (MIC) and minimum bactericidal concentration (MBC) of CLEO against *P. gingivalis* were determined by broth microdilution. Anti-biofilm activity was assessed via XTT assay. Network pharmacology, molecular docking, and 100 ns molecular dynamics simulations were employed to identify active compounds and core targets. Experimental periodontitis was induced in C57BL/6 mice by molar ligation. Mice received topical CLEO at concentrations of 2, 3, and 4 mg/mL, 2% minocycline, or vehicle once daily for 14 days. Periodontal inflammation, alveolar bone loss, collagen organization, osteoclast activity, and serum levels of MMP-9 and COX-2 were evaluated. CLEO exhibited potent anti-*P. gingivalis* activity, with an MIC of 2 mg/mL and MBC of 4 mg/mL. At the MIC, CLEO disrupted 57.5% of pre-formed *P. gingivalis* biofilms. Network pharmacology and molecular docking identified α-bisabolol, chamazulene, and 1,8-cineole as key active compounds, with the chamazulene-HSP90AA1 complex showing the strongest binding affinity (−10.0 kcal/mol). The 100 ns MD simulation confirmed the stability of this complex (RMSD < 1 nm). In the mouse periodontitis model, topical application of CLEO at 3 and 4 mg/mL significantly reduced gingival inflammation, alveolar bone resorption, and the number of TRAP-positive osteoclasts compared with the vehicle-treated periodontitis group (all *p* < 0.05). Furthermore, CLEO treatment dose-dependently lowered serum MMP-9 levels (from 24.15 ± 0.24 pg/mL in the model group to 12.36 ± 0.54 pg/mL in the high-dose group) and COX-2 levels (from 15.38 ± 0.62 pg/mL to 8.99 ± 0.57 pg/mL). The therapeutic efficacy of the high-dose CLEO group was comparable to that of the 2% minocycline group. CLEO exerts anti-*P. gingivalis* and anti-biofilm effects in vitro and ameliorates periodontitis in vivo through multi-target mechanisms, providing pharmacological evidence for its traditional use in inflammatory conditions.

## 1. Introduction

Periodontitis, a chronic inflammatory disease driven by dysbiotic plaque biofilms, affects a substantial portion of the global population, with its unequal distribution strongly shaped by socioeconomic determinants [1]. In addressing this public health challenge, a joint EFP/ORCA consensus report has previously outlined integrated strategies for the prevention and control of both dental caries and periodontitis at individual and population levels, emphasizing the importance of shared risk factor management [2]. Beyond the oral cavity, the disease exhibits bidirectional associations with systemic conditions. Evidence from a recent review of systematic reviews with meta-analyses and a separate systematic review demonstrates that non-surgical periodontal treatment significantly reduces glycated hemoglobin in patients with type 2 diabetes [3,4]. Furthermore, a systematic review and meta-analysis of observational studies have identified a significant link between periodontitis and the risk of Alzheimer’s disease [5]. The underlying mechanisms involve direct hematogenous spread of periodontal pathogens, systemic dissemination of inflammatory mediators, and common immuno-inflammatory pathways that fuel comorbid diseases [6].

Current treatment of periodontitis relies primarily on mechanical debridement and adjunctive antibiotics, aiming to restore oral microbial homeostasis [7]. However, these conventional approaches are increasingly challenged by incomplete biofilm removal and the alarming rise in antimicrobial resistance, which substantially limit their long-term efficacy [7,8]. To overcome such limitations, diverse modern antimicrobial materials and strategies have been investigated. Carbonaceous nanoparticles represent one explored route, exhibiting antibacterial activity against oral pathogens, yet their intraoral long-term safety and cytotoxic potential remain a concern [9]. Locally delivered antibiotics can achieve high gingival crevicular fluid concentrations, but the widespread dissemination of antibiotic resistance genes among subgingival microbiota-including *Porphyromonas gingivalis*-seriously undermines their clinical reliability [10]. Phage therapy and predatory bacteria have been proposed as highly specific alternatives capable of lysing periodontal pathogens, and recent studies further elucidate how phages shape the periodontal microbial ecosystem, providing a basis for potential therapeutic manipulation [7,11]. In parallel, natural polyphenolic compounds have garnered considerable attention. For instance, honokiol combined with resveratrol has been shown to inhibit oral bacteria responsible for malodor and their biofilm [12], while selected dietary polyphenols directly suppress the growth and biofilm formation of key periodontopathogens such as *P. gingivalis* [13]. Recent reviews further highlight the promise of polyphenols in combating multidrug-resistant periodontal pathogens and their underlying molecular mechanisms [14,15]. In the realm of hybrid materials, functionalized cyclotriphosphazene-modified dental compositions have been developed for 3D printing of dental crowns, representing advanced material engineering for dental applications [16]. Although these strategies each hold promise, they collectively face obstacles related to biocompatibility, synthetic complexity, high cost, or biological stability.

In this context, plant-derived essential oils have drawn increasing attention as a compelling natural alternative. Essential oils offer distinct advantages: multi-component synergism that may delay the development of resistance, a long history of traditional use supporting their safety, and straightforward topical application without the need for elaborate fabrication [17]. For example, lemongrass essential oil has been reported to exert multi-target inhibitory effects on *P. gingivalis* by coordinately regulating heme utilization, biofilm formation, and ferroptosis-related metabolic pathways [17]. Despite such progress, many valuable essential oil resources remain unexplored, highlighting the need to identify novel candidates that combine robust anti-biofilm efficacy with a favorable safety profile.

*Chrysanthemum lavandulifolium* (Fisch. ex Trautv.) Makino (Asteraceae), commonly known as “Gan Ju” in China, is a perennial herb widely distributed across Qinghai, Gansu, Shaanxi, and Xinjiang, where it grows on mountain slopes, rocky terraces, river valleys, and loess hills [18]. Traditional Chinese medicine records indicate that the plant has long been used to clear heat, eliminate toxins, reduce swelling, cool the liver, and improve vision. Its inflorescence is applied for carbuncles, boils, and bloodshot eyes, and the aerial parts are used for clearing heat, detoxifying, and treating headache, red eyes, and pneumonia. These heat-clearing and swelling-relieving properties are closely aligned with the typical “oheat toxin” manifestations of periodontitis, namely red, swollen, and painful gingiva.

Despite this traditional background, the essential oil of *C. lavandulifolium* (CLEO) has not been systematically investigated for its anti-periodontitis potential. Modern pharmacological research has shown that Chrysanthemum species possess notable anti-inflammatory and antioxidant activities, and the essential oils derived from them exhibit significant antibacterial and anti-inflammatory effects [19,20,21]. These findings provide a compelling rationale to evaluate the efficacy of CLEO against periodontitis. Therefore, this study aimed to comprehensively assess the antimicrobial and therapeutic effects of CLEO on *Porphyromonas gingivalis* (*P. gingivalis*) and experimental periodontitis in mice. Specifically, we (1) determined the antibacterial and anti-biofilm activities of CLEO against *P. gingivalis* in vitro; (2) employed network pharmacology, molecular docking, and molecular dynamics simulations to identify key active components and core targets; and (3) evaluated the in vivo effects of CLEO on periodontal inflammation, alveolar bone resorption, and systemic inflammatory markers in a mouse periodontitis model. This work provides the first pharmacological evidence supporting the use of *C. lavandulifolium* for gingival and periodontal health, and explores its potential as a novel natural therapeutic agent for periodontitis.

## 2. Results

### 2.1. In Vitro Antibacterial and Anti-Biofilm Activities of CLEO

**Figure 1:**
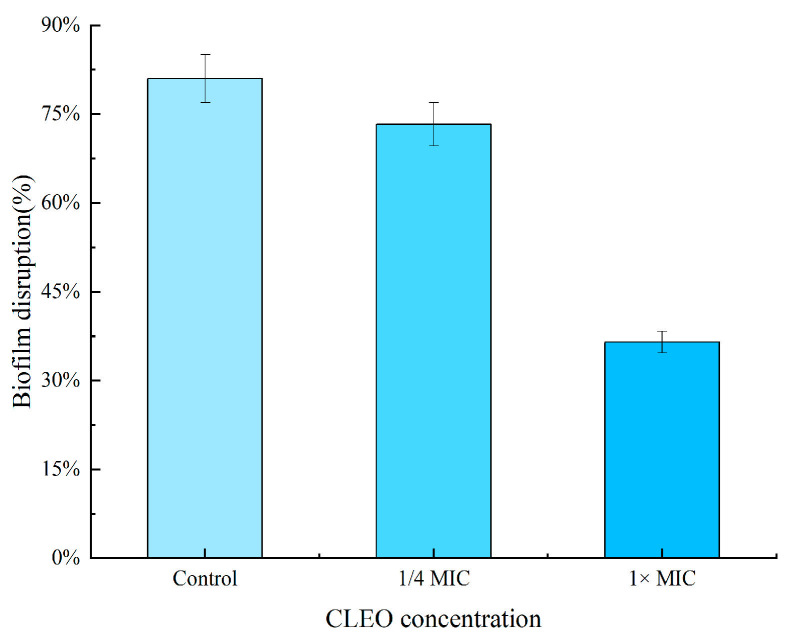
Anti-biofilm activity of CLEO against pre-formed *P. gingivalis* biofilms. Data are expressed as mean ± SD (*n* = 3).

The MIC and MBC values of CLEO against *P. gingivalis* were determined to be 2 mg/mL and 4 mg/mL, respectively. In the anti-biofilm assay, CLEO disrupted pre-formed *P. gingivalis* biofilms in a dose-dependent manner. At a concentration of 2 mg/mL (1× MIC), the biofilm eradication rate reached 57.5% (*p* < 0.05). In contrast, the 0.5 mg/mL (1/4× MIC) group showed no significant difference compared with the untreated control group (*p* > 0.05). These quantitative results are presented in Figure 1.

### 2.2. Network Pharmacology and Molecular Simulation

#### 2.2.1. Active Ingredients and Target Identification

**Figure 2:**
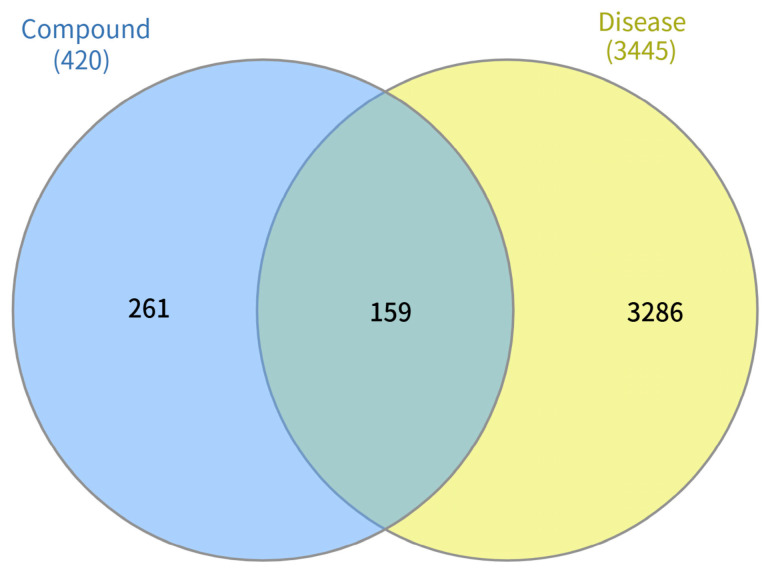
Venn diagram showing the intersection of potential targets of CLEO active ingredients and periodontitis-related disease targets.

Five active ingredients of CLEO (α-bisabolol, nootkatone, chamazulene, trans-nerolidol, and 1,8-cineole) were identified. After target prediction and deduplication, 261 potential targets were linked to these components. By intersecting them with 3286 periodontitis-associated disease targets, 159 common targets were identified as potential therapeutic targets of CLEO against periodontitis (Figure 2).

#### 2.2.2. PPI Network and Core Target Analysis

**Figure 3:**
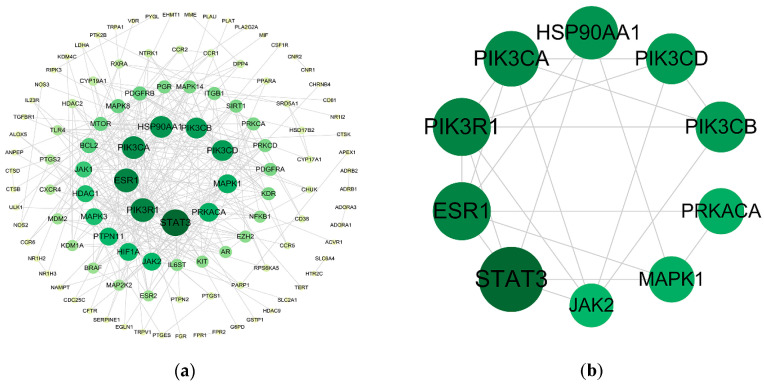
Protein–protein interaction (PPI) network of the 159 overlapping targets. (**a**) Full PPI network; node size reflects degree centrality. (**b**) Top-ranked core targets; darker color indicates higher composite score.

A PPI network was constructed from the 159 intersecting targets. Topological analysis identified STAT3, HSP90AA1, ESR1, PIK3R1, and PIK3CA as the top five core targets based on their degree, betweenness, and closeness centrality values (Figure 3a,b).

#### 2.2.3. GO and KEGG Enrichment

**Figure 4:**
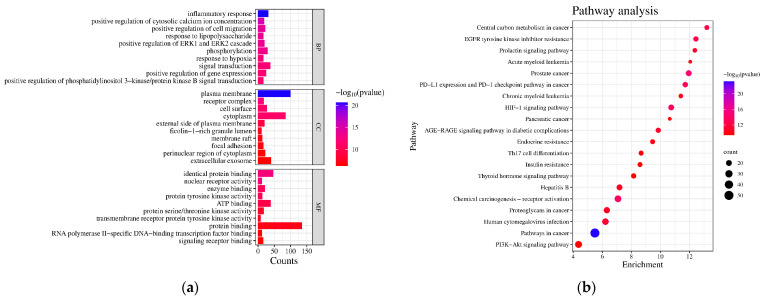
GO and KEGG enrichment analyses. (**a**) GO enrichment bubble chart (top 10 terms in BP, CC, MF). (**b**) KEGG pathway enrichment bubble chart (top 20 pathways). Bubble color represents −log10(*p*-value); bubble size indicates gene count (**a**) or gene ratio (**b**).

GO enrichment analysis showed that the intersecting targets were mainly associated with biological processes such as the inflammatory response and positive regulation of cell migration. KEGG analysis revealed their involvement in pathways including EGFR tyrosine kinase inhibitor resistance and the HIF-1 signaling pathway, suggesting that multi-pathway regulation is key to the anti-periodontitis effects of CLEO (Figure 4a,b).

#### 2.2.4. Molecular Docking

**Figure 5:**
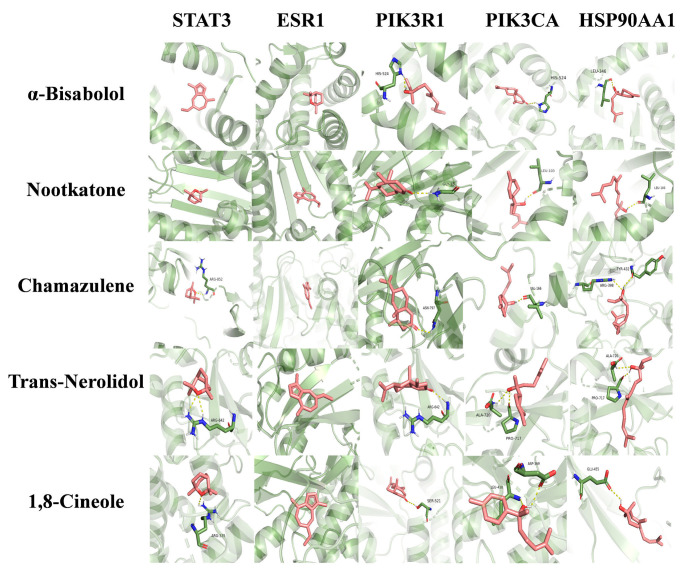
Docking pose of chamazulene within the HSP90AA1 binding pocket. Hydrogen bonds are shown as dashed lines.

Molecular docking demonstrated that all five active components bound stably to the top five core targets, with all binding energies below −5.0 kcal/mol (Table 1). The chamazulene-HSP90AA1 complex exhibited the lowest binding energy, and its docking conformation is shown in Figure 5.

#### 2.2.5. Molecular Dynamics (MD) Simulation

**Figure 6:**
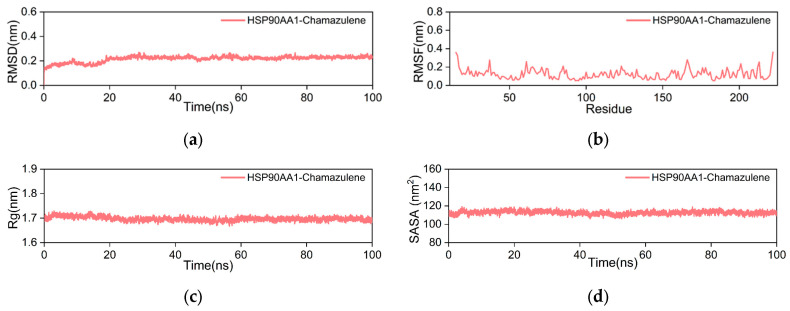
Molecular dynamics simulation of the HSP90AA1-chamazulene complex (100 ns). (**a**) Root mean square deviation (RMSD). (**b**) Root mean square fluctuation (RMSF). (**c**) Radius of gyration (Rg). (**d**) Solvent accessible surface area (SASA).

**Figure 7:**
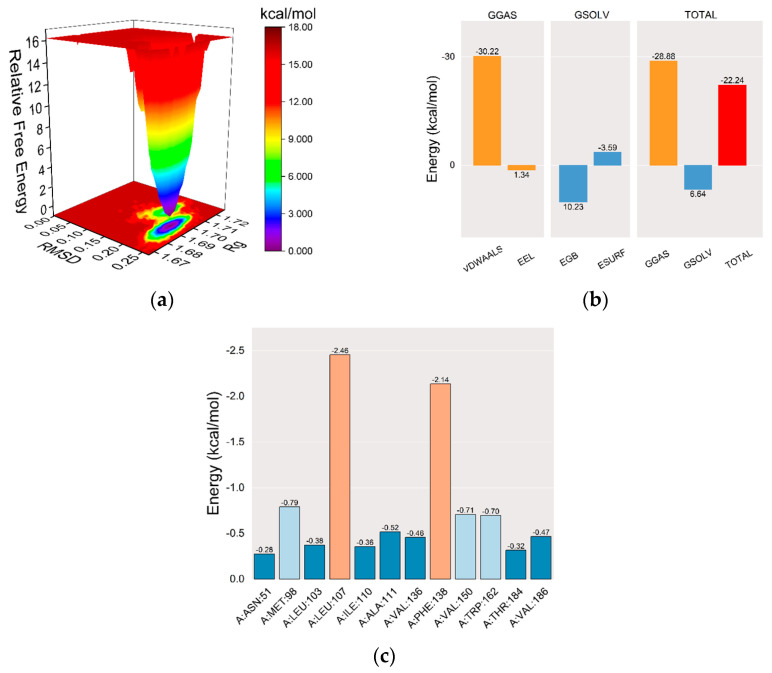
Free energy analysis of the HSP90AA1-chamazulene complex. (**a**) Free energy landscape (FEL). (**b**) Binding free energy components (MM/GBSA). (**c**) Per-residue energy contributions of key amino acids in HSP90AA1.

**Figure 8:**
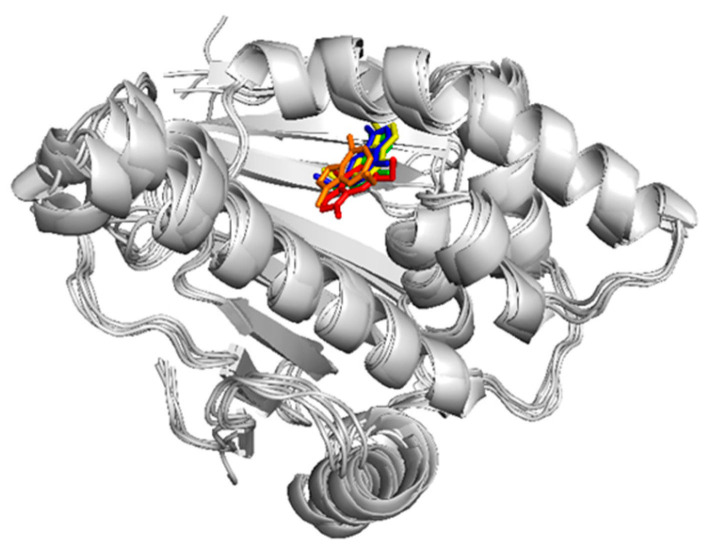
Superimposed conformations of the HSP90AA1-chamazulene complex at 0, 25, 50, 75, and 100 ns. Chamazulene is colored red, green, blue, yellow, and orange, respectively.

The chamazulene-HSP90AA1 complex was subjected to 100 ns MD simulations. The RMSD curve remained below 1 nm (Figure 6a), and RMSFs were minimal (Figure 6b). The Rg and SASA values remained stable throughout the simulation (Figure 6c,d). The free energy landscape (FEL) exhibited a single, concentrated minimum energy basin, confirming high complex stability (Figure 7a). MM/GBSA calculations yielded a total binding free energy of −22.24 kcal/mol, with LEU107 and PHE138 identified as the key residues contributing most to binding (Figure 7b,c). Conformational alignment at five time points (0, 25, 50, 75, and 100 ns) confirmed that chamazulene maintained a highly stable binding pose within the HSP90AA1 pocket (Figure 8).

### 2.3. Animal Experiment Results

#### 2.3.1. Establishment of the Mouse Periodontitis Model

**Figure 9:**
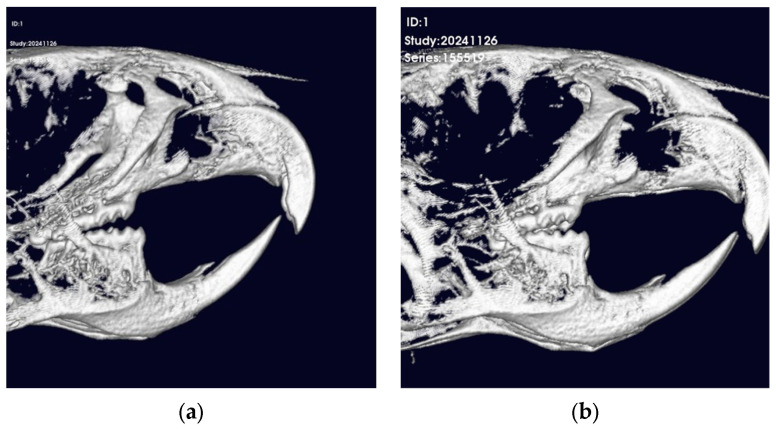
Micro-CT images of the maxillary first molar region. (**a**) Normal control (NG) group. (**b**) Experimental periodontitis (EP) group at day 7 post-ligation.

**Figure 10:**
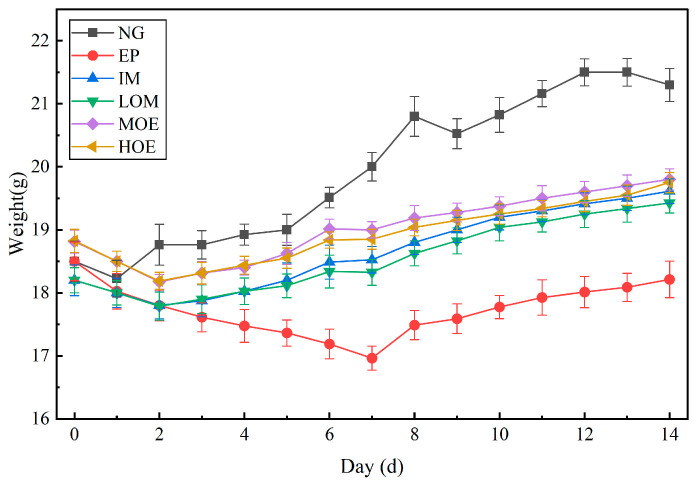
Changes in body weight of mice in each group during the 14-day experimental period. Data are expressed as mean ± SD (*n* = 8). No significant differences were found among groups at any time point.

Micro-CT imaging at day 7 post-ligation confirmed successful periodontitis induction, with a visibly increased distance from the alveolar bone crest to the cementoenamel junction (CEJ) in the EP group compared with the NG group (Figure 9a,b). Body weight changes over the 14-day period are shown in Figure 10.

#### 2.3.2. Histopathological Analysis

**Figure 11:**
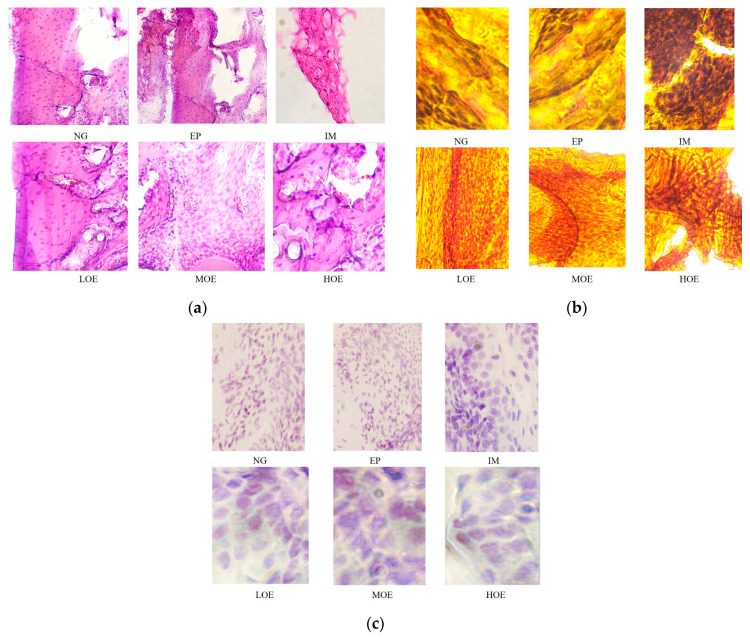
Histopathological analysis of periodontal tissues (original magnification ×200). (**a**) H&E staining showing inflammatory infiltration and alveolar bone loss. (**b**) Sirius red staining under polarized light for collagen fiber organization. (**c**) TRAP staining for osteoclasts (red, multinucleated cells). NG, normal control; EP, experimental periodontitis; IM, 2% minocycline; LOE, 2 mg/mL CLEO; MOE, 3 mg/mL CLEO; HOE, 4 mg/mL CLEO.

H&E staining showed that CLEO treatment, particularly in the MOE (3 mg/mL) and HOE (4 mg/mL) groups, markedly alleviated gingival epithelial destruction, inflammatory cell infiltration, and alveolar bone resorption compared with the EP group. Sirius red staining under polarized light confirmed improved collagen fiber density and alignment in CLEO-treated groups. TRAP staining revealed a significant, dose-dependent reduction in osteoclast numbers following CLEO treatment, with the HOE group achieving efficacy comparable to that of the minocycline (IM) group. These histological findings are summarized in Figure 11a–c.

#### 2.3.3. Serum Inflammatory Factor Levels

As shown in Table 2, serum MMP-9 and COX-2 levels were significantly elevated in the EP group compared with the NG group (*p* < 0.05). The MOE and HOE groups significantly downregulated both factors (*p* < 0.05 vs. EP), with no statistically significant difference between the HOE and IM groups (*p* > 0.05).

## 3. Discussion

This study provides the first systematic evidence that *Chrysanthemum lavandulifolium* essential oil (CLEO) exerts multi-target therapeutic effects against periodontitis. Specifically, we demonstrated that (1) CLEO potently inhibited *P. gingivalis* (MIC = 2 mg/mL) and disrupted pre-formed biofilm in a dose-dependent manner; (2) network pharmacology combined with molecular docking and 100 ns MD simulations identified STAT3, HSP90AA1, EGFR, and HIF-1 pathway components as core targets, with chamazulene exhibiting stable binding to HSP90AA1; and (3) in a mouse model of ligature-induced periodontitis, locally applied CLEO (3–4 mg/mL) significantly attenuated alveolar bone loss, reduced osteoclast activity, and downregulated serum MMP-9 and COX-2 levels, achieving efficacy comparable to that of 2% minocycline. These findings provide the first pharmacological validation of the traditional use of *C. lavandulifolium* for oral inflammatory conditions.

CLEO exhibited potent antibacterial activity against *P. gingivalis* and disrupted pre-formed biofilm in a dose-dependent manner. This finding is consistent with the growing body of evidence that plant-derived essential oils and bioactive compounds can inhibit periodontal pathogens [15]. Notably, a recent study on *Satureja montana* and *Leptospermum scoparium* essential oils demonstrated multi-mode antibacterial mechanisms against *P. gingivalis*, including membrane damage and suppression of virulence factors [22]. The concentration-dependent anti-biofilm effect of CLEO mirrors the behavior reported for other natural agents, such as ellagic acid, punicalagin, and quercitrin, which interfere with *P. gingivalis* and *Fusobacterium nucleatum* biofilms [23,24,25]. Moreover, the necessity of targeting *P. gingivalis* is underscored by the fact that specific polymorphic variants of its peptidylarginine deiminase gene are associated with enhanced pathogenicity [26], highlighting the value of antimicrobial strategies against this keystone pathogen.

Network pharmacology analysis identified STAT3 and HSP90AA1 as core targets through which CLEO components may exert anti-periodontitis effects. STAT3 signaling is involved in osteoblast differentiation and inflammatory bone remodeling [27], and pharmacological modulation of the JAK2/STAT3 pathway has been shown to attenuate periodontal tissue damage in a rat periodontitis model [28]. HSP90AA1 encodes the stress-inducible chaperone HSP90α and was previously identified as a key hub gene in peri-implantitis [29]. Silencing HSP90AA1 in *P. gingivalis* lipopolysaccharide-stimulated human gingival fibroblasts significantly reduces inflammatory cytokine release and oxidative stress, an effect linked to the restoration of autophagy [30]. These prior findings substantiate our computational prediction that chamazulene binds stably to HSP90AA1, thereby modulating downstream inflammatory cascades.

KEGG pathway enrichment revealed that the core targets are enriched in the HIF-1 signaling pathway. HIF-1α is now recognized as a pivotal contributor to periodontal tissue destruction, as comprehensively reviewed by Fadl and Leask [31]. Under hypoxic conditions, *P. gingivalis* synergistically amplifies HIF-1α and NF-κB activation in periodontal ligament cells, fueling a pro-inflammatory feedback loop [32]. The predicted interaction of CLEO components with this pathway provides a mechanistic rationale for the observed reduction in both local inflammation and systemic inflammatory markers.

In vivo, locally applied CLEO (3–4 mg/mL) significantly reduced gingival epithelial destruction, inflammatory infiltration, and alveolar bone loss in a dose-dependent manner. These histological improvements were paralleled by a marked decrease in TRAP-positive osteoclasts and downregulation of serum MMP-9. MMP-9 is a key extracellular matrix protease that accelerates periodontal tissue breakdown; notably, MMP-9 deficiency has been shown to paradoxically accelerate periodontitis progression by impairing tissue repair, underscoring its complex role [33]. The concomitant reduction in serum COX-2 levels further supports the systemic anti-inflammatory effect of CLEO. Importantly, the efficacy of 4 mg/mL CLEO was statistically comparable to that of 2% minocycline, highlighting its translational potential as a natural therapeutic agent or adjunct.

This study has several limitations. The essential oil was obtained by steam distillation, whereas traditional preparations use aqueous decoction; comparative pharmacological studies are warranted. The absence of an independent acute toxicity assessment is a limitation of this study; future studies should systematically evaluate the acute toxicity of CLEO in the same animal model to determine the safe dose range. The molecular mechanisms were inferred from network pharmacology and in silico simulations and require direct validation through binding assays, gene silencing, or pathway-specific inhibitors. Additionally, the local pharmacokinetics, long-term safety, and effects on the commensal oral microbiota remain to be investigated. Future studies should prioritize these aspects, along with formulation optimization and randomized controlled trials, to fully establish the clinical utility of CLEO in periodontitis management.

## 4. Materials and Methods

### 4.1. Reagents and Instruments

*Chrysanthemum lavandulifolium* essential oil (CLEO) was provided by the Key Laboratory of Medicinal Animal and Plant Resources of the Qinghai–Tibet Plateau and was extracted via steam distillation. Prior to this study, the chemical composition of CLEO was analyzed by our group using GC-MS, which identified terpenoids as the predominant chemical class (32.92%), with chamazulene (2.64%), trans-nerolidol (1.25%), and α-bisabolol (0.84%) among the major constituents [34]. *Porphyromonas gingivalis* (ATCC 33277) was obtained from Beijing BioDee Biotechnology Co., Ltd, Beijing, China. BHI broth, hemin, vitamin K1, and minocycline hydrochloride were acquired from Shanghai Yuanye Bio-Technology Co., Ltd., Shanghai, China. The XTT assay kit was sourced from Hunan Aikerui Bioengineering Co., Ltd., Changsha, China. Staining kits were procured from Beijing Solarbio Science & Technology Co., Ltd., Beijing, China, and ELISA kits were obtained from Wuhan Huamei Biotech Co., Ltd., Wuhan, China. Key instruments included an anaerobic culture system (Mitsubishi Gas Chemical, Japan Mitsubishi Gas Chemical, Tokyo, Japan), a microplate reader (ReadMax 1200, Shanghai Shanpu, Shanghai, China), a micro-CT system (Quantum GX2, PerkinElmer, Waltham, MA, USA), and standard histological equipment.

### 4.2. Determination of MIC, MBC, and Anti-Biofilm Activity

*P. gingivalis* was cultured in BHI broth supplemented with hemin (0.5%) and vitamin K1 (1%) at 37 °C under anaerobic conditions for 48 h. Anaerobic conditions were achieved using an anaerobic jar (Mitsubishi Gas Chemical, Tokyo, Japan) with AnaeroPack™ anaerobic gas generating sachets (Mitsubishi Gas Chemical, Japan), which produce an atmosphere of approximately 0.1% O_2_, 10–15% CO_2_, and balanced N_2_. An anaerobic indicator strip was used to verify the maintenance of anaerobic conditions throughout the incubation period.

The MIC and MBC of CLEO against *P. gingivalis* were determined by broth microdilution following the Clinical and Laboratory Standards Institute (CLSI) guidelines for anaerobic bacteria [35]. CLEO was serially diluted (1–8 mg/mL) in BHI broth containing 0.5% DMSO. A bacterial suspension (1 × 10^7^ CFU/mL) was added, and the mixtures were incubated anaerobically at 37 °C for 48 h. The MIC was defined as the lowest concentration at which no visible bacterial growth was observed. For MBC determination, aliquots from wells showing no growth were subcultured onto BHI blood agar plates and incubated anaerobically at 37 °C for 48 h. The MBC was defined as the lowest concentration yielding ≤5 colonies, in accordance with the same guideline [35].

For biofilm experiments, BHI broth supplemented with 0.5% hemin and 1% vitamin K1 was used as the growth and biofilm formation medium throughout. To evaluate the dose-dependent anti-biofilm activity of CLEO, two concentrations were tested: 1× MIC (2 mg/mL) and 1/4× MIC (0.5 mg/mL). The 1× MIC represents the minimum inhibitory concentration of CLEO against *P. gingivalis*. The sub-MIC (1/4× MIC) was included to determine whether CLEO exhibits anti-biofilm activity at concentrations below the bactericidal threshold and to confirm the presence of a dose–response relationship.

Biofilm formation was assessed in 96-well plates using BHI broth supplemented with 0.5% hemin and 1% vitamin K1, which was the same growth medium used for bacterial subculture. A *P. gingivalis* suspension (1 × 10^6^ CFU/mL) was added to the plates and allowed to adhere for 4 to 24 h. After the removal of the supernatant and washing with PBS, fresh medium containing CLEO (0.5, 1, or 2 mg/mL) was added, and the plates were incubated for an additional 24 h. Biofilm formation and metabolic activity were assessed using the XTT reduction assay originally described by Roehm et al. (1991) [36], with minor modifications for bacterial biofilm experiments. The percentage of biofilm eradication was calculated based on the following standard formula, where the untreated control group was defined as 100% biofilm formation [37]:

$$Biofilm\;eradication\;rate\;(\%)\;=\;\frac{{OD}_{control}\;-\;{OD}_{treated}}{{OD}_{control}}\;\times \;100\%$$

where OD_control_ is the absorbance of the untreated control group and OD_treated_ is the absorbance of the CLEO-treated group. Each experiment was performed in triplicate with three parallel wells per group.

### 4.3. Network Pharmacology and Molecular Simulation

Active components of CLEO were screened using the TCMSP database (https://tcmsp.91medicine.cn/#/home, accessed on 10 July 2024) based on the criteria of oral bioavailability ≥ 30% and drug-likeness ≥ 0.18, and further verified through literature review. Compound targets were predicted using SwissTargetPrediction (http://swisstargetprediction.ch/, accessed on 10 July 2024). Periodontitis-related targets were retrieved from GeneCards (https://www.genecards.org, accessed on 10 July 2024), OMIM (https://www.omim.org, accessed on 10 July 2024), and DisGeNET. The intersecting targets were imported into STRING (version 12.0, confidence score > 0.9, https://cn.string-db.org/) to construct a protein–protein interaction (PPI) network. Core targets were identified using Cytoscape 3.9.1 based on degree centrality [38]. Gene Ontology (GO) and Kyoto Encyclopedia of Genes and Genomes (KEGG) enrichment analyses were performed using DAVID database (https://davidbioinformatics.nih.gov/, accessed on 10 July 2024) and Metascape platform (https://metascape.org, accessed on 10 July 2024). Results were visualized using the online bioinformatics platform (https://www.bioinformatics.com.cn, accessed on 10 July 2024).

Molecular docking was performed using AutoDockTools 1.5.7. Protein structures were obtained from the Protein Data Bank (PDB) (STAT3: 6NJS; HSP90AA1: 1UYC) (https://www.rcsb.org, accessed on 10 July 2024), and compound structures were retrieved from PubChem. The complex with the lowest binding energy was selected for 100 ns molecular dynamics (MD) simulations using GROMACS 2022.4 at 310 K and 1 bar. The root-mean-square deviation (RMSD), root-mean-square fluctuation (RMSF), radius of gyration (Rg), and solvent-accessible surface area (SASA) were analyzed.

The selection of these five terpenoids was based on the following rationale. First, prior GC-MS analysis of CLEO (Section 4.1) identified terpenoids as the predominant chemical class, with chamazulene, trans-nerolidol, and α-bisabolol among the major constituents. Second, all five compounds satisfied the TCMSP screening criteria (oral bioavailability ≥ 30%, drug-likeness ≥ 0.18). Third, their antimicrobial and anti-inflammatory properties are well-documented: α-bisabolol attenuates inflammation via downregulation of MAPK and NF-κB signaling [39]; chamazulene, an azulene derivative, exhibits broad anti-inflammatory activity [40]; trans-nerolidol has been shown to exert anti-inflammatory effects in human primary leukocytes [41]; 1,8-cineole possesses antibacterial and antioxidant activities [42]; and nootkatone displays antifungal activity, indicative of broad antimicrobial potential [43]. Accordingly, these five terpenoids were prioritized as representative bioactive components of CLEO for network pharmacology and molecular docking analyses.

### 4.4. Mouse Model of Periodontitis

All animal experiments were approved by the Animal Ethics Committee of Qinghai Normal University (Approval No. 2026-091, approved on 12 September 2024). The study was conducted in accordance with the National Guidelines for the Care and Use of Laboratory Animals (GB/T 35892-2018) [44] and the ARRIVE guidelines. Every effort was made to minimize animal suffering in accordance with the 3R principles. All surgical procedures were performed under anesthesia with 10% chloral hydrate, and all animals were humanely euthanized by cervical dislocation under deep anesthesia at the end of the experiment.

Forty-eight female C57BL/6 mice were randomly divided into six groups (*n* = 8): normal control (NG), experimental periodontitis (EP), 2% minocycline (IM), and CLEO at 2, 3, and 4 mg/mL (low-dose, LOE; medium-dose, MOE; high-dose, HOE). Under chloral hydrate anesthesia, periodontitis was induced by ligating the maxillary first molar with a 5-0 silk suture. A volume of 20 μL of each treatment was locally injected into the gingival sulcus daily for 14 consecutive days. After sacrifice, serum was collected for measurement of MMP-9 and COX-2 levels by enzyme-linked immunosorbent assay (ELISA). The maxillae were fixed, decalcified, embedded in paraffin, and sectioned. Tissue sections were subjected to hematoxylin and eosin (H&E) staining, Sirius red staining (observed under polarized light microscopy), and tartrate-resistant acid phosphatase (TRAP) staining. Osteoclasts were identified as TRAP-positive multinucleated cells (≥3 nuclei) and counted.

### 4.5. Statistical Analysis

Data are presented as the mean ± standard deviation (SD). Statistical analyses were performed using SPSS 23.0. A *p*-value < 0.05 was considered statistically significant.

## 5. Conclusions

This study shows that *Chrysanthemum lavandulifolium* essential oil (CLEO) exerts potent antibacterial and anti-biofilm activities against *Porphyromonas gingivalis* (MIC = 2 mg/mL; biofilm eradication rate of 57.5% at 2 mg/mL) and effectively ameliorates experimental periodontitis in mice. Network pharmacology and in silico analyses point to multi-target mechanisms involving STAT3, HSP90AA1, EGFR, and HIF-1 pathways, and molecular docking together with 100 ns molecular dynamics simulations confirm that the active components, particularly chamazulene, bind stably to HSP90AA1. In vivo, locally applied CLEO at 3–4 mg/mL significantly reduced periodontal inflammation, preserved alveolar bone, and downregulated serum MMP-9 and COX-2 levels, with efficacy comparable to that of 2% minocycline. Importantly, these findings provide the first pharmacological evidence supporting the traditional use of *C. lavandulifolium* for clearing heat, reducing swelling, and treating inflammatory conditions of the gingiva and periodontal tissues. CLEO therefore emerges as a promising natural multi-target candidate for adjunctive periodontitis therapy and merits further clinical investigation.

## Figures and Tables

**Table 1:** Binding free energy (kcal/mol) between effective compounds in CLEO and key target proteins.

Compound	STAT3	ESR1	PIK3R1	PIK3CA	HSP90AA1
α-Bisabolol	−7.0	−7.4	−5.2	−7.9	−8.4
Nootkatone	−7.5	−8.2	−5.6	−7.4	−8.1
Chamazulene	−6.8	−7.9	−5.8	−7.7	−10.0
trans-Nerolidol	−6.2	−7.1	−5.9	−7.2	−7.8
1,8-Cineole	−5.7	−6.0	−6.1	−5.7	−5.6

**Table 2:** Serum levels of MMP-9 and COX-2 in each experimental group.

Group	COX-2 (pg/mL)	MMP-9 (pg/mL)
NG	6.69 ± 0.44 *	10.32 ± 0.28 *
EP	15.38 ± 0.62 △	24.15 ± 0.24 △
IM	9.21 ± 0.77 *△	12.58 ± 0.39 *△
LOE	12.17 ± 0.36 *△	19.31 ± 0.62 *△
MOE	9.39 ± 0.92 *△	15.75 ± 0.38 *△
HOE	8.99 ± 0.57 *△	12.36 ± 0.54 *△
F	81.098	526.416
*p*	<0.001	<0.001

Note: Data are expressed as mean ± SD (*n* = 8). NG, normal control; EP, experimental periodontitis; IM, 2% minocycline; LOE, 2 mg/mL CLEO; MOE, 3 mg/mL CLEO; HOE, 4 mg/mL CLEO. F and *p* values were obtained from one-way ANOVA. * *p* < 0.05 vs. NG group; △ *p* < 0.05 vs. EP group. No significant difference was observed between the HOE and IM groups for either marker (*p* > 0.05).

## Data Availability

The original contributions presented in this study are included in the article. Further inquiries can be directed to the corresponding author.

## Abbreviations

The following abbreviations are used in this manuscript:

CLEO	*Chrysanthemum lavandulifolium* Essential Oil
COX-2	Cyclooxygenase-2
DMSO	Dimethyl Sulfoxide
EGFR	Epidermal Growth Factor Receptor
ESR1	Estrogen Receptor 1
HIF-1	Hypoxia-Inducible Factor-1
MBC	Minimum Bactericidal Concentration
MIC	Minimum Inhibitory Concentration
MMP-9	Matrix Metalloproteinase-9
*P. gingivalis*	*Porphyromonas gingivalis*
PBS	Phosphate Buffered Saline
PD	Periodontal Disease
RMSD	Root Mean Square Deviation
RMSF	Root Mean Square Fluctuation
SASA	Solvent Accessible Surface Area
STAT3	Signal Transducer and Activator of Transcription 3
